# The Advancement of Targeted Alpha Therapy and the Role of Click Chemistry Therein

**DOI:** 10.3390/molecules30061296

**Published:** 2025-03-13

**Authors:** Sara Lacerda, Robin M. de Kruijff, Kristina Djanashvili

**Affiliations:** 1 Centre de Biophysique Moléculaire, CNRS UPR 4301, Université d’Orléans, Rue Charles Sadron, 45071 Orléans, France; sara.lacerda@cnrs.fr; 2 Department of Radiation Science and Technology, Delft University of Technology, Mekelweg 15, 2629 JB Delft, The Netherlands; r.m.dekruijff@tudelft.nl; 3 Department of Biotechnology, Delft University of Technology, Van der Maasweg 9, 2629 HZ Delft, The Netherlands

**Keywords:** pre-targeting, targeting vectors, tumors, recoiling daughters, complexation, click chemistry, targeted alpha therapy, radionuclides, chelators

## Abstract

Recent years have seen a swift rise in the use of α-emitting radionuclides such as ^225^Ac and ^223^Ra as various radiopharmaceuticals to treat (micro)metastasized tumors. They have shown remarkable effectiveness in clinical practice owing to the highly cytotoxic α-particles that are emitted, which have a very short range in tissue, causing mainly double-stranded DNA breaks. However, it is essential that both chelation and targeting strategies are optimized for their successful translation to clinical application, as α-emitting radionuclides have distinctly different features compared to β^−^-emitters, including their much larger atomic radius. Furthermore, upon α-decay, any daughter nuclide irrevocably breaks free from the targeting molecule, known as the recoil effect, dictating the need for faster targeting to prevent healthy tissue toxicity. In this review we provide a brief overview of the current status of targeted α-therapy and highlight innovations in α-emitter-based chelator design, focusing on the role of click chemistry to allow for fast complexation to biomolecules at mild labeling conditions. Finally, an outlook is provided on different targeting strategies and the role that pre-targeting can play in targeted alpha therapy.

## 1. Introduction

The effect of radiation on the human body has interested scientists since the discovery of X-rays. Modern nuclear medicine puts a lot of effort into the development of targeted radionuclide therapy (TRT), which in oncology is considered a promising application for the treatment of various types of cancers based on the use of radiolabeled drugs that specifically target molecular pathologies [1]. In contrast to external beam radiotherapy (EBRT), which is mainly applied to a specific area, the advantage of TRT is seen in its ability to target and treat metastatic cancers. This comes from the fact that in TRT, the radioisotope is injected intravenously, reaching the whole body while circulating, as opposed to EBRT, where the beam is focused on a particular body region. TRT may also play an important role in the palliative care of cancer patients for whom treatment is no longer an option [2] and holds potential for the treatment of viral and bacterial infections [3].

Many radioisotopes have been explored for medical applications, with their utility determined by their decay type, energy, and half-life. These characteristics dictate their suitability for either imaging and/or therapeutic purposes. Diagnostic radionuclides typically emit gamma (γ) rays or positrons (β^+^), which are ideal for imaging, while therapeutic radionuclides are selected for their strong interaction with matter and limited tissue penetration, commonly involving alpha (α), beta-minus (β^−^), or Auger electron emissions. Among these, β^−^ particles have the longest range (generally ≤12 mm), followed by α-particles with ranges of 50–100 µm, equivalent to less than 10 cell diameters, and, finally, Auger electrons have the shortest range with a pathlength of only 2–500 nm, limiting their impact to single cells. In addition to differences in the pathlength, radioisotopes also vary in their linear energy transfer (LET)—a measure of the energy deposited into tissue per unit of distance. Alpha-emitting radionuclides exhibit the highest LET, reaching 80–100 keV/µm, followed by Auger electrons at approximately 4–26 keV/µm, and β^−^-particles at around 0.2 keV/µm [4]. Considering these main features of radionuclides in terms of their biological use, Auger electron emitters often need precise targeting to the tumor cell nucleus to maximize their efficacy, while β^−^-emitters are more suitable for treating medium-to-large tumors but carry a higher risk of collateral damage to surrounding healthy tissue due to their longer range.

**Figure 1:**
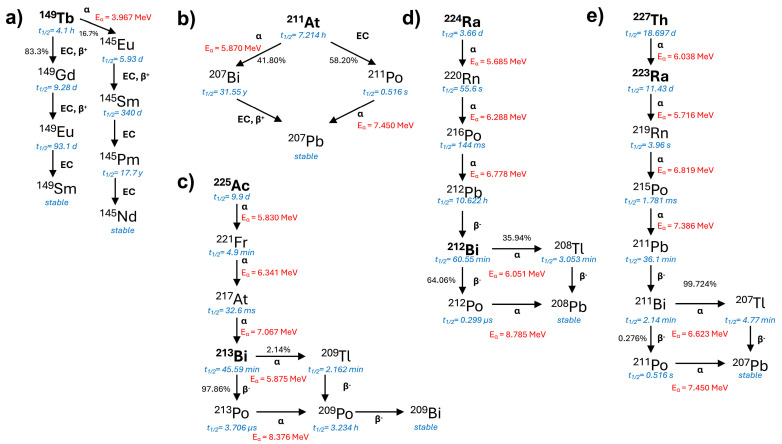
Characteristics of the main α-emitting radionuclides of medical interest, including their respective half-lives (blue) and main alpha energy (red), based on nuclear data from Live Chart of Nuclides [20]: (**a**) ^149^Tb [21], (**b**) ^211^At [22], (**c**) ^225^Ac and ^213^Bi [23,24,25], (**d**) ^224^Ra and ^212^Bi [26], and (**e**) ^227^Th and ^223^Ra [23,27].

Compared to β^−^-emitting radionuclides, α-emitters with their short pathlengths and high LET are significantly more cytotoxic. Their high-energy emissions predominantly cause double-stranded DNA breaks and DNA cluster damage, which are more challenging for cells to repair [5]. Moreover, α-emitters are effective regardless of the tumor cell cycle or oxygenation status, making them particularly advantageous for targeting resistant tumor cells. It has been estimated that achieving a single cell kill probability of 99.99% requires tens of thousands of β^−^-decays, whereas only a few α-decays at the cell membrane are sufficient to reach the same level of cytotoxicity [6,7]. These distinctive biophysical properties of α-emitters (Figure 1) have driven significant interest in their use in targeted alpha therapy (TAT), which shows particular promise for treating small neoplasms or micrometastases [8,9,10]. Beyond their potent direct cytotoxicity, α-emitters may also cause additional biological interactions, such as the induction of immune responses and the bystander effect [11]. These considerations emphasize the critical importance of achieving high-precision delivery of α-emitters to tumor tissues through smart targeting strategies to advance TAT into clinical practice. However, even with highly efficient targeting mechanisms, a persistent challenge is the recoil of α-emitting daughters from the radiopharmaceutical, which leads to undesired radiation exposure [12]. Briefly, after emitting an α-particle, daughter radionuclides gain a recoil energy, typically between 100 and 200 keV, which is orders of magnitude higher than the energy of a chemical bond (a few eV) and, therefore, always resulting in bond rupture, allowing them to break free from the radiocomplex. Although these recoiled daughter nuclides travel a distance of only about 100 nm in tissue [13], when the radiopharmaceutical is still circulating in the blood, this results in their rapid dispersion throughout the body, as they are highly unlikely to rebind with the original chelator. As a result, the recoiled daughters cease to target tumor tissue and instead accumulate in healthy organs, posing a severe toxicity risk. Clearly, addressing the challenge of retaining α-emitting daughters at the target site requires advanced strategies, but it is equally crucial to ensure the rapid in vivo biodistribution of TAT-agents in addition to the development of high-precision targeted delivery routes.

The high tumor affinity of antibodies has established them as the gold standard targeting vectors in TRT despite the known accompanying disadvantages, such as slow pharmacokinetics, limiting their application to long-lived radionuclides. Attempts to solve this problem, e.g., by engineered antibodies, including the use of their fragments or small peptide moieties, have unfortunately resulted in reduced tumor accumulation [14]. To circumvent these problems, developments over the past decade have focused on pre-targeting approaches, which essentially involve sequential administration of a targeting vector that is allowed to accumulate in the tumor, followed by a radioisotope bound to a bifunctional chelator (BFC), modified with a moiety that is primed to react with the preadministered vector [15]. Reactions serving this strategy can basically be divided into four categories: (1) streptavidin–biotin [16], (2) bispecific antibodies [17], (3) oligonucleotides [18], and (4) click chemistry [19], presented in comprehensive reviews on radiochemistry using pre-targeting approaches in general and each of the methodologies in particular. Here, we focus on the challenges for the advancement of TAT to clinical application and the role of click chemistry in this process, specifically in the synthesis of α-emitting radiopharmaceuticals and their rapid delivery to tumors via a bio-orthogonal pre-targeting mechanism.

## 2. Targeted Alpha Therapy in Clinical Application

**Figure 2:**
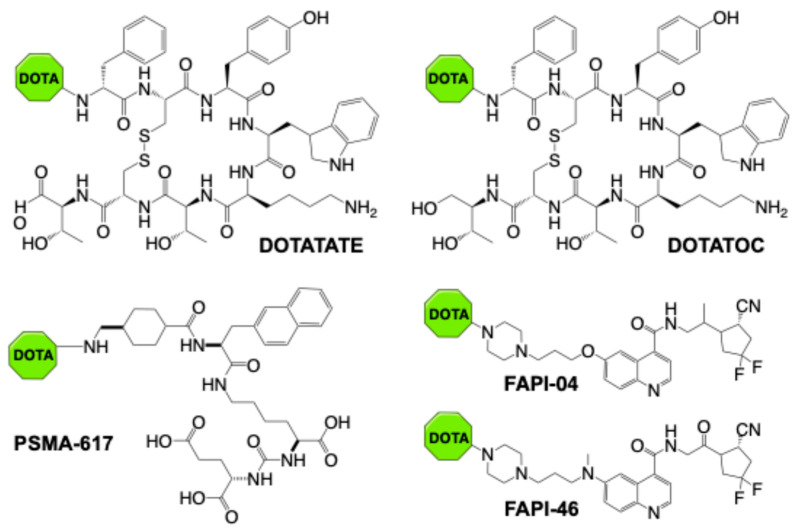
Structures of the bifunctional chelators considered for clinical applications in combination with α-emitters.

Although the clinical potential of ^223^Ra was already recognized in the early 1940s [28], its unchelated form, ^223^RaCl_2_ (Xofigo^®^; Bayer HealthCare, Leverkusen, Germany, approved in 2013), remains the only clinically approved α-emitting radiopharmaceutical. It naturally targets the bone hydroxyapatite matrix, leveraging its affinity for areas of active bone remodeling such as those affected by metastatic cancer [29]. Despite many other TAT agents currently undergoing clinical trials, the most prominent examples of modern TRT still concern β^−^-emitting radiopharmaceuticals, which have shown remarkable results and received clinical approval, including [^153^Sm]Sm–lexidronam (Quadramet^®^, CIS Bio International, Gif-sur-Yvette, France), [^131^I]I–iobenguane (Azedra^®^, Progenics Pharmaceuticals, Inc., New York, NY, USA), or antibody-based [^90^Y]Y–ibritumomab tiuxetan (Zevalin^®^, CIS Bio International, Gif-sur-Yvette, France). More recently, [^177^Lu]Lu–DOTATATE (Lutathera™, Advanced Accelerator Applications, Rueil-Malmaison, France) and [^177^Lu]Lu–PSMA-617 have been introduced, with the latter radiopharmaceutical specifically developed for the treatment of metastatic castration-resistant prostate cancer (mCRPC) based on targeting the prostate-specific membrane antigen (PSMA). Interestingly, the development of α-radiotherapeutic agents often follows the path of adapting chelators originally developed for β^−^-radiotherapy (Figure 2) by substituting the radionuclides and optimizing treatment planning protocols [7,9]. The most noteworthy examples are [^225^Ac]Ac–PSMA-617 and [^225^Ac]Ac–DOTATATE, both of which are currently undergoing clinical trials and demonstrate very promising clinical outcomes [30,31]. In these cases, the adaptation of β^−^-based radiopharmaceuticals to TAT agents allowed for the direct comparison of therapeutic efficacy between the different types of emitters. A case study demonstrated the remarkable therapeutic efficacy of [^225^Ac]Ac–PSMA-617 in achieving complete remission for a clinically critical patient previously unresponsive to a [^177^Lu]Lu–PSMA-617 treatment [32]. Similarly, a recent study by Ballal et al. revealed that a significant number of patients with stable or progressive disease after a [^177^Lu]Lu–DOTATATE therapy achieved partial remission following treatment with [^225^Ac]Ac–DOTATATE [33].

Other examples of successful repurposing of existing radiopharmaceuticals for TAT are [^213^Bi]Bi– and [^225^Ac]Ac–DOTA–Substance P. Both demonstrated encouraging clinical results for the treatment of glioblastoma multiforme [34], while initial studies were conducted with the β^−^-emitters ^90^Y and ^177^Lu [35]. Similarly, the developed somatostatin analog DOTATOC was initially labeled with β^−^-emitters such as ^90^Y and ^177^Lu, and later tested in patients resistant to β^−^-DOTATOC therapy by using the α-emitter ^213^Bi analogue, showing a good therapeutic outcome [36]. More recently, a shift has been observed where newly developed targeting agents utilize both β^−^- and α-emitters from the outset. For instance, following the development of several fibroblast activation protein inhibitors (FAPI) for PET imaging of fibroblasts associated with many cancers [37], the development of β^−^- and α-emitter-based FAPI radiopharmaceuticals has been going hand in hand. Lindner et al. achieved promising results for a significant reduction in analgesic effects with [^90^Y]Y–FAPI-04 in the treatment of two patients with metastasized breast cancer without observable side effects [38], which was quickly followed by an investigation by Watabe et al. on the effectiveness of [^225^Ac]Ac–FAPI-04 in mice [39]. The relatively rapid tumor washout of FAPI-04 prompted the development of FAPI-46 with a higher tumor uptake and a much slower clearance, which was studied with both ^177^Lu and ^225^Ac [40]. Interestingly, a direct comparison of [^177^Lu]Lu–FAPI-46 and [^225^Ac]Ac–FAPI-46 revealed a similar therapeutic efficacy for both radionuclides; however, [^177^Lu]Lu–FAPI-46 demonstrated longer-lasting effects and superior in vivo stability [41]. This difference may be particularly relevant given that FAPIs target cancer-associated fibroblasts rather than cancer cells directly, making the short range of α-particles from [^225^Ac]Ac–FAPI-46 potentially insufficient for complete tumor eradication.

Although many different α-emitting radionuclides have been identified and studied, only a few have the desired characteristics that render them suitable for clinical use [7,10]. The interested reader is referred to one of several recent reviews [42,43,44,45,46] which discuss in detail the status of α-emitting radiopharmaceuticals in clinical application, but which are beyond the scope of this review.

## 3. Considerations in Bifunctional Chelator Design for α-Emitters

A common feature among developed radiopharmaceuticals is their reliance on a chelating agent, paired with a tumor-specific targeting moiety. While the role of targeting moieties is to ensure the specificity of the radiopharmaceutical for certain cancer cells, the choice of the chelator should in principle align with the properties of the radionuclide. It is also important to consider the chemical form in which it is available to ensure optimal radiolabeling efficiency, i.e., a rapid production of radioactive complexes with quantitative yields and under mild conditions (ambient temperature, pH close to neutral), compatible with the typical targeting groups (peptides or antibodies) that make up BFCs. This ideal concept is, however, rarely realized and, as some studies suggest, the strategy of repurposing chelators and targeting agents for TAT faces several challenges, some of which require more attention than they currently receive in research [47].

**Figure 3:**
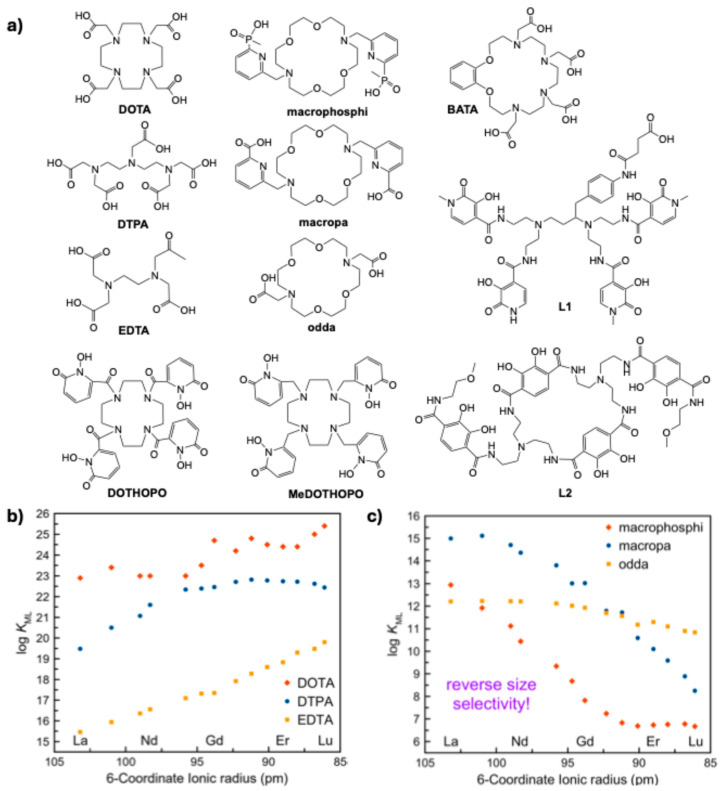
Chemical structures of selected chelators (**a**) and thermodynamic formation stability constants (log*K*_ML_) measured after complexation with the various Ln^3+^ ions plotted vs. ionic radii (**b**,**c**), adopted from [58].

In addition to the higher energy released during α-decay and the recoil phenomenon, another distinguishing feature between α- and β^−^-emitters is their atomic size. The “gold standard” DOTA chelator (Figure 3a) and its derivatives are widely used for chelating a variety of trivalent metal ions, including lanthanides, which typically exhibit high thermodynamic stability constants [48]. Within the lanthanide series, contraction of the *f*-orbitals causes a reduction in the ionic radius [49], with La (106.1 pm) being a larger ion than Lu (84.8 pm). In contrast, medically relevant α-emitting radiometals (except for ^149^Tb) have much larger atomic radii (e.g., Ac—112 pm, Bi—117 pm, Pb—120 pm, or Ra—162 pm) compared to the commonly used β^−^-emitters. This size difference allows for the direct analysis of how the ionic size impacts the stability of metal complexes, leading to the conclusion that (1) larger radiometals require chelators with appropriately sized cavities to ensure greater thermodynamic and kinetic stability and (2) chelators designed for smaller metal ions are less effective at retaining larger ions in vivo, resulting in reduced complex stability and potentially increased healthy tissue toxicity [41].

Price and Orvig already reported the relevance of matching chelators to the radiometal of choice a decade ago [47], and research on this topic keeps growing. For example, the thermodynamic stability of a series of chelators (Figure 3a) as a function of different metal ion sizes clearly demonstrates that macrocyclic types of chelators provide more stable complexes compared to open-chain ones (e.g., DOTA vs. DTPA, EDTA) (Figure 3b) and that larger cavity chelators do a better job fitting α-emitting radiometals (e.g., macropa, macrophosphi, and odda vs. DOTA) (Figure 3c). This highlights the critical importance of designing chelators specifically tailored for α-emitting radiometals and emphasizes that DOTA, due to its thermodynamic stability being inversely related to the ionic radius of the metal ion, is a relatively poor chelator for α-emitters such as ^225^Ac [50], as evidenced by the reduced stability of the [^225^Ac]Ac–DOTA complex in vivo [51]. A significant contribution in this direction has recently been made by Ivanov et al., who combined experimental and computational data to study the complexation behavior of Ra^2+^ with two macrocycles, DOTA and macropa, demonstrating the superior chelating properties of the latter compound [52].

Due to the lack of stable isotopes of actinium, lanthanum is typically used as a suitable nonradioactive surrogate thanks to their chemical similarity, namely their ionic radius. Standard potentiometric titrations for thermodynamic stability determination require a substantial amount of ligand and metal, making it difficult to use alternative methods to access Ac complexes directly. Nevertheless, Kalmycov et al. have proposed a method based on extraction, which was validated via a comparison with La complexes (using ICP-MS as a detection method for La as opposed to γ-counting for ^225^Ac). They studied both DOTA and BATA chelators (Figure 3a) and revealed that BATA yields Ac complexes with superior stability, with log*K* affinities of 20.3 for DOTA and 25.7 for BATA. Moreover, radiolabeling BATA with ^225^Ac was possible at room temperature and neutral pH, advocating the interest in this ligand, which could also be functionalized via the benzene ring [53].

Novel ligands designed for the complexation of thorium were also reported, consisting of a large octadentate open-chain structure with four Me-3,2-HOPO arms (L1 [54]), a macrocycle with two terephthalamide pendant arms (L2 [55]), or macrocyclic chelators bearing 1,2-hydroxypyridinone groups (DOTHOPO and MeDOTHOPO [56]) (Figure 3a). All these complexes exhibited a remarkably high thermodynamic stability, with log*K* values between 34 and 53.

Many of the long-lived α-emitting radionuclides currently under investigation for TAT, such as ^225^Ac and ^223^Ra, have multiple α-emitting daughter nuclides in their decay chains, which significantly increases their cytotoxicity due to the recoil effect. Minimizing the distribution of these highly toxic daughter nuclides to healthy organs requires a rapid delivery of the α-emitting radiopharmaceutical to the target site, ideally followed by its internalization into tumor cells. In contrast, β^−^-emitters, which do not experience the recoil effect [57], may be better suited for slower-targeting agents such as antibodies, depending on their half-life. Finally, as previously noted, α-emitters have a much higher LET and shorter range than β^−^-emitters, making them generally more effective for treating smaller (micro)metastases, which in turn necessitates different targeting strategies.

## 4. Bioconjugation Strategies and the Role of Click Chemistry

A critical aspect of the development of BFCs as radiopharmaceuticals for TAT is the establishment of a covalent chemical linkage between the radiometal–chelator complex and the tumor targeting vector, typically a biomolecule such as a monoclonal antibody or peptide. As large and sensitive entities, antibodies can be affected by harsh reaction conditions that may lead to changes in their chemical structure and, consequently, to a reduction in their targeting specificity. For the same reason, the conjugation point must be carefully chosen, prearranging the location of the established links to avoid the formation of heterogeneous products with inconsistent pharmacokinetics. Finally, all chemical modifications must take into account the possible immunogenic effects caused by the presence of non-native chemical bonds, which basically implies the preference for biocompatible linkers along with mild reaction conditions. Spicer et al. have reviewed the key strategies for achieving controlled conjugation of biomolecules, highlighting the relative advantages and disadvantages of each technique [59], with the most common reactions being based on amines and thiols.

**Scheme 1:**
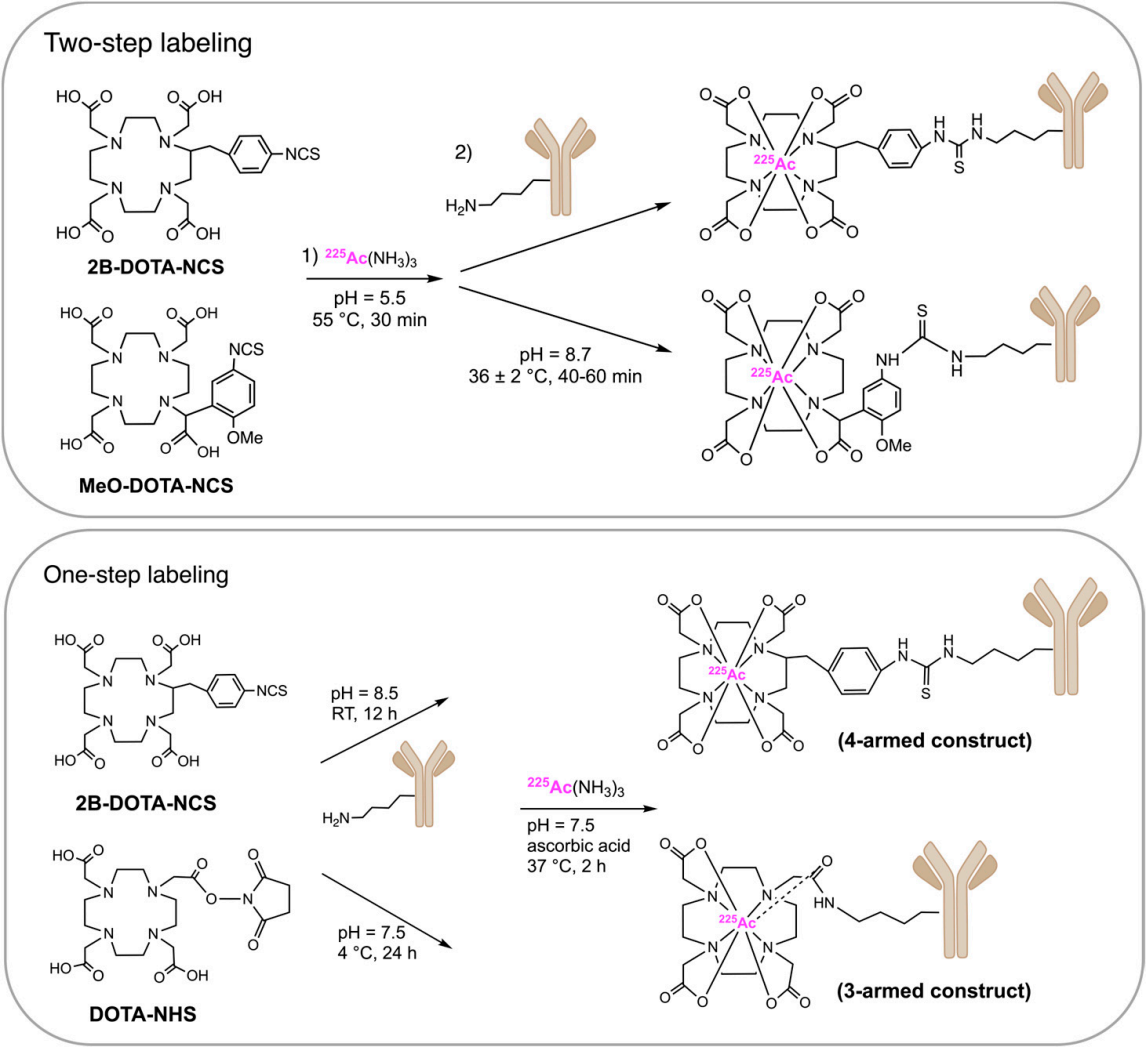
Two-step and one-step labeling protocols for ^225^Ac antibody derivatives. Adapted from [60,62].

In the first example of [^225^Ac]Ac–DOTA conjugation to a tumor-targeting antibody, a two–step process was employed, starting from DOTA functionalized with an isothiocyanate (NCS) attached to either the macrocycle backbone (2B–DOTA–NCS) or to the ethylene group (MeO–DOTA–NCS) (Scheme 1) to avoid disruption of coordination with the radiometal [60]. Both chelators were first radiolabeled with ^225^Ac following a typical DOTA complexation procedure (≈55 °C, pH 5, 30 min) and then conjugated to a series of monoclonal antibodies through a reaction between lysine residues and NCS groups, forming stable urea bonds, with the best results obtained for J591 (anti-PSMA) [60]. The same bioconjugation strategy was applied to prepare [^225^Ac]Ac–DOTA–trastuzumab for the SKOV3 ovarian tumor model to treat micrometastases through intraperitoneal injection of the α-emitting radiopharmaceutical [61]. Nevertheless, this two–step labeling protocol led to a rather low radiochemical global yield (7–17%) as approximately 90% of the actinium was coordinated by nonreactive forms of DOTA in the first step of the procedure, being subsequently discarded, thus lowering the overall yield. Next, the authors investigated a direct one–step labeling of preformed antibody DOTA-like constructs [62], achieving a 10-fold higher radiochemical yield (>80%) and a 30-fold higher specific activity, while performing the reaction under mild conditions, i.e., with a reaction temperature of 37 °C. In the same study, a comparison was made between DO3A-based derivatives in which the lysine residue of the antibody was conjugated to one of the carboxylic arms via an NHS group and DOTA-based chelators functionalized with an NCS group on the macrocycle backbone. Again, it could be concluded that although the two conjugates led to quite stable radiocomplexes, both the kinetics of labeling and the binding capacity for ^225^Ac appeared to be greater for the 4-armed construct compared to the 3-armed derivative (see Scheme 1 for the coordination). In the 3-armed derivative, the oxygen atom of the amide group also coordinates, but in a less stable manner. These observations confirm once more that a larger number of donor atoms, or stronger bonded ones, leads to better stability of α-emitting radiocomplexes.

**Scheme 2:**
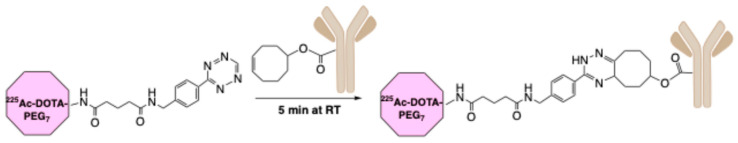
Click reaction proposed for radiolabeling of a TCO-functionalized antibody with [^225^Ac]Ac–DOTA–Tz [63].

To overcome the low yield of two–step labeling procedures and enhance purification, click chemistry can be utilized to conjugate the biomolecule to a preformed radiocomplex. This approach permits the use of harsh conditions during radiolabeling while preserving the integrity of the biomolecule’s targeting moiety. As click reactions are typically fast and clean, they are particularly well-suited for the efficient production of radiopharmaceuticals. Hence, in the context of radiopharmaceutical development, click chemistry has been widely applied, both for synthesizing the chelator itself and for conjugating biomolecules to radiocomplexes. An illustrative example of an improved synthesis of the [^225^Ac]Ac–DOTA–antibody conjugate was reported by Poty et al., who used radiolabeled DOTA functionalized with tetrazine (Tz) in an inverse electron-demand Diels–Alder reaction (IEDDA) with a *trans*-cyclooctene (TCO)-conjugated antibody [63] (Scheme 2). This method not only outperformed the conventional NCS approach [60], as reflected in a superior radiochemical yield of the bioconjugate product formed within 5 min under milder conditions, but also demonstrated the modular potential of this reaction.

**Figure 4:**
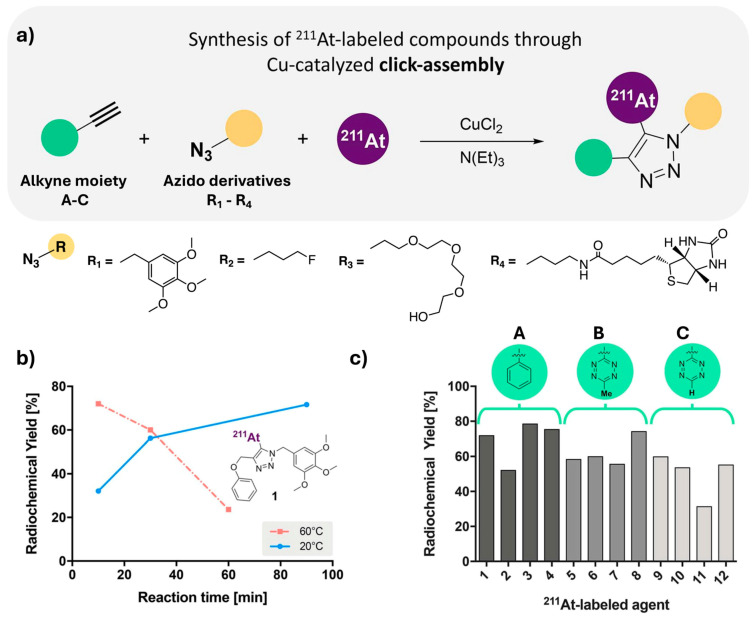
An example of a modular assembly of radiopharmaceuticals: (**a**) schematic representation of a one–pot copper-catalyzed click reaction, (**b**) the radiochemical yield as a function of reaction time, (**c**) comparison of radiochemical yields (10 min, 60 °C) of compounds composed of azido derivatives (yellow circle) and alkyne moieties (green circle): **1–4** = A + R_1_–R_4_; **5–6** = B + R_1_–R_4_; and **7–12** = C + R_1_–R_4_. Adopted from [64].

Alpha-emitting radiopharmaceuticals have also been prepared via “one–pot click” reactions. Denk et al. reported on a ^211^At-based radiopharmaceutical that appeared to be more stable in vivo compared to its dehalogenated counterparts when prepared via a click reaction. A CuAAC (copper-catalyzed azide–alkyne cycloaddition) conjugation was employed in the presence of an α-emitting ^211^At ion, which was generated through oxidation by the Cu catalyst. The astatine ion underwent electrophilic substitution with a triazole ring, resulting in the formation of 5-[^211^At]At-1,2,3-triazole products. A study with a modular library of 12 compounds, combining alkyne moieties A to C with four azido derivatives R_1_–R_4_ (Figure 4a), reported radiochemical yields of 70%. Notably, similar radiochemical yields of approximately 70% were achieved under two distinct conditions: high temperature (60 °C) with a short reaction time (10 min) and low temperature (20 °C) with a longer reaction time (90 min) (Figure 4b) [64].

**Figure 5:**
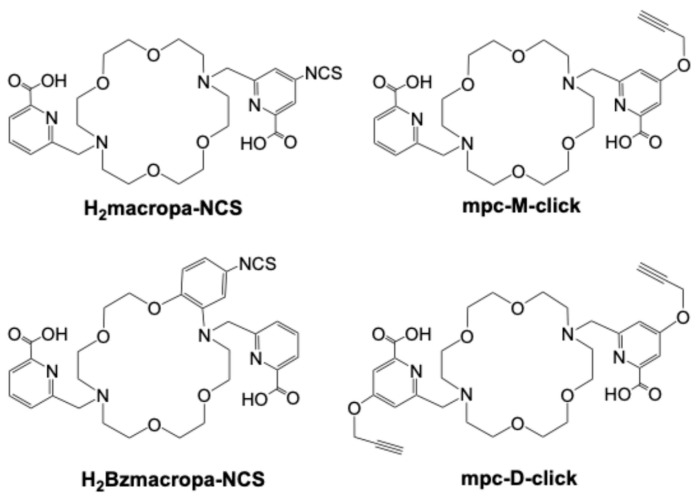
Chelators pre-functionalized for bioconjugation with α-emitting radiocomplexes with tumor-targeting vectors.

Later on, PSMA derivatives of ^225^Ac were synthesized using click reactions. Reissig et al. proposed macropa derivatives functionalized with one (mcp-M-click) or two (mcp-D-click) propyne units as replacements for the NCS group on the picolinate pendant arm(s) of the ligand H_2_–macropa–NCS (Figure 5). Using CuAAC ligation, they attached one or two azide-bearing PSMA-targeting vectors to the chelator core. After labeling with ^225^Ac, the authors observed that the bivalent probe exhibited a nearly 9-fold higher binding in vitro than its monovalent counterpart and demonstrated prolonged tumoral retention in a murine model of prostate cancer [65]. Zeglis et al. have recently revisited the click chemistry toolbox over the last two decades [66]. They have provided an overview of areas where the use of click chemistry has become quite essential in the field of radiopharmaceutical sciences, including automation of labeling processes, site-specific bioconjugations, multimerization, probe purification, and in vivo pre-targeting, all of which contribute to the improvement of clinical nuclear medicines.

## 5. Pre-Targeting as a Delivering Strategy in TAT

Special delivery strategies need to be developed for TAT to focus the α-radiation dose on malignant tissue to damage its DNA and suppress its repair while sparing healthy tissue. At the cellular level, cancer cells differ from healthy cells primarily by the overexpression of certain receptors. An ideal receptor is located at the cell surface without being released into the bloodstream and is prone to binding agonist/antagonist molecules. Such binding often triggers the internalization process through a so-called receptor-mediated mechanism. Therefore, targeting tumor cells requires specialized vectors with an affinity for these receptors that can be coupled to a moiety carrying an α-emitting radionuclide, as is the case with TAT. On the other hand, the tumor resides in a particular microenvironment that exhibits unique characteristics, such as weak and leaky blood vessels, high acidity and low oxygen levels, the presence of immune cells, etc. [67]. Consideration of these parameters, either as independent or auxiliary targeting elements, may improve treatment efficacy even if receptor availability/overexpression is not optimal [68]. In fact, the exploitation of these synergistic strategies may lead to tailored radiotherapies that profit from both tumor specificity and the surrounding conditions.

The effectiveness of radiolabeled antibodies as targeting vectors has already been demonstrated in radioimmunoimaging. However, classical antibody-based approaches to radiotherapy face significant challenges, including: (1) slow pharmacokinetics leading to prolonged blood circulation times (up to 5 days), causing undesired radiation exposure to healthy organs, (2) limited mobility of large antibody molecules (~150 kDa) within the tumor tissue resulting in inefficient penetration of radiotherapeutics into the tumor core, (3) impaired interaction with the immune system reducing therapeutic efficacy, and (4) low overall tumor uptake, which remains a critical limitation. Peptides have been proposed as an alternative to antibodies, offering faster kinetics and better tumor penetration due to their smaller size, but their targeting precision is limited.

**Figure 6:**
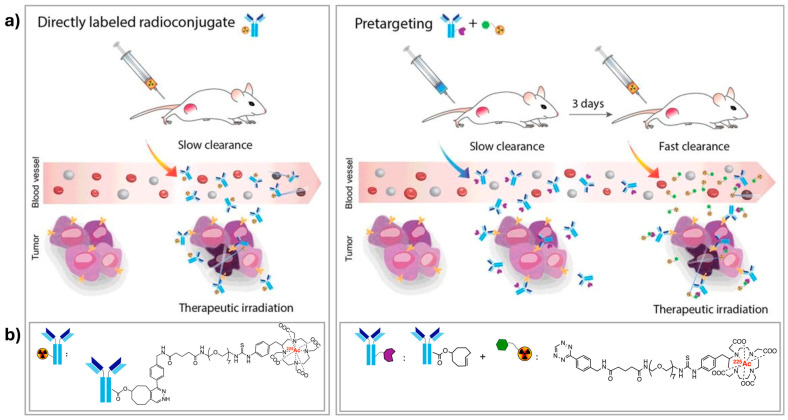
(**a**) Schematic representation of two administration routes for radiopharmaceuticals: direct administration of radioligands (**left**) and two–step administration of chemically modified targeting vectors (e.g., antibodies), followed by radioligands provided with matching functional groups designed for a click reaction (**right**); (**b**) radiopharmaceuticals involved in the corresponding study [75].

Since its introduction in the late 1980s, pre-targeting has been recognized as a promising alternative to address the aforementioned challenges [69]. In vivo pre-targeting has undergone significant development over the last decade, leading to a wealth of research and comprehensive reviews on the topic [70,71]. However, despite this progress, there is only a handful of studies exploring its potential application in alpha therapy. Generally, in radiotherapy, pre-targeting involves the physical separation of the targeting vector and the radiolabel component, requiring a two–step administration procedure. First, the targeting molecule is injected and allowed to accumulate at the site of interest, followed by the injection of a small radiocomplex designed to bind specifically to the targeting molecule (Figure 6a). The waiting time between these two steps is typically adapted to the kinetics of the targeting molecule. In some cases, a clearing agent is administered after the initial step to actively remove the excess targeting vector still circulating in the bloodstream [72]. The obvious general advantage of pre-targeting is the reduction of radiation exposure to healthy organs and tissues. However, its effectiveness depends on the availability of the targeting vector on the surface of tumor cells so it can bind with the radiocomplex, as well as on the high selectivity and efficiency of the reaction between the two components.

Several approaches have been proposed to implement the concept of pre-targeting by utilizing various in vivo binding mechanisms between targeting molecules and complexes. These include the use of bispecific antibodies (BsAbs) with dual affinity for specific tumor antigens and radiolabeled hapten molecules [73], strept(avidin)–mAb bioconjugates that noncovalently bind radiolabeled biotin [65], and the hybridization of complementary oligonucleotides, where one is conjugated to an antibody and the other to a radioligand [74]. Finally, the assembly of targeting molecules and radioligands in vivo can be achieved by means of a click reaction between the two components, both of which are pre-equipped with suitable functional groups.

Compared to the other aforementioned pre-targeting methods, the strategy based on click reactions is characterized by remarkably fast kinetics and high specificity. The latter is due to the bio-orthogonal nature of the reaction, which occurs exclusively between the radiocomplex and the pre-targeted molecules, thereby reducing off-target interactions. While the classical click reaction CuAAC cannot be applied in vivo due to the involvement of a toxic copper catalyst, its counterparts—IEDDA (inverse electron-demand Diels–Alder reaction, *k*_2_ > 10^3^ M^−1^ s^−1^) and SPAAC (strain-promoted azide–alkyne cycloaddition, *k*_2_~0.1 M^−1^ s^−1^) are being intensively investigated for pre-targeted delivery of radiopharmaceuticals. One of the earliest examples of in vivo biorthogonal pre-targeting utilized an IEDDA reaction between an electron-deficient tetrazine-based [^111^In]In radiocomplex suitable for SPECT (single photon emission computed tomography) imaging and a chemically modified antibody (CC49) conjugated to a strained *trans*-cyclooctene (TCO) [76]. Mice bearing colon carcinoma xenografts were first injected with CC49-TCO, followed by the administration of an ^111^In–tetrazine complex one day later. This approach resulted in significant accumulation of the small radiocomplex at the tumor site, unlike in animals preinjected with unmodified antibodies. In 2019, Poty et al. reported a comparative study of a similar pre-targeting strategy [75] using an ^225^Ac-labeled tetrazine radioligand and a TCO-bearing anti-CA19.9 antibody (5B1) for pancreatic ductal adenocarcinoma versus conventional radioimmunotherapy with a directly ^225^Ac-radiolabeled immunoconjugate (Figure 6b). The results confirmed the superiority of the pre-targeting approach in reducing off-target toxicity while delivering radiotherapeutic payloads to the tumor. Interestingly, the authors utilized Cerenkov radiation from ^225^Ac’s daughter decay to visualize the biodistribution both in vivo and postmortem. This provided critical insights into the behavior of ^225^Ac-based radiopharmaceuticals, valuable for their further (pre)clinical development.

In addition to the need for preclinical evaluations to demonstrate the potential of click chemistry in pre-targeted α-radiotherapy, significant efforts are being made to chemically optimize the design of both reactants: targeting molecules and radiocomplexes. The synthetic considerations concern the correlation of click efficiency, reactivity, lipophilicity of tetrazines, and stability [77]. Recently, poly-l-lysine effector molecules of two sizes (21 and 10 kDa) functionalized on both sides with prosthetic groups for radiohalogenation (^211^At) and tetrazine attachment were presented and evaluated in healthy Balb/C mice [78]. The molecules demonstrated a good stability regardless of their size, and their blood clearance matched the 7.2-h half-life of ^211^At.

## 6. Conclusions and Further Outlook

Despite its remarkable therapeutic value, α-radionuclide therapy is still underrepresented in clinical settings. In addition to practical barriers, such as the availability of α-emitters, costs, and production/distribution logistics, its development is also hindered by chemical challenges related to designing novel chelates specific to α-emitting radiometals, as well as biological challenges. These include the high energy, short path length, recoiling and bystander effects of α-emitting radionuclides, which place demands on an extremely accurate and rapid delivery to the site of interest.

An exciting frontier in TAT development lies in the use of bio-orthogonal click reactions, which can serve as powerful tools for both the synthesis of radiocomplexes and their pre-targeted delivery to tumors—both aspects are explored in this review. In the synthesis of radiocomplexes, click chemistry offers high efficiency and, most importantly, compatibility with mild conditions necessary for bioconjugation with targeting moieties. The versatility of the click reaction enables modular libraries of radiocomplexes and targeting vectors that can be mixed and matched depending on the envisioned radionuclide and the defined tumor target. On the other hand, the bio-orthogonal nature of the click reaction makes it ideally suited for the delivery of highly toxic α-radiopharmaceuticals through the decoupling of targeting and radiolabeling steps, thereby reducing systemic toxicity and enhancing therapeutic indices.

There is no doubt that the role of click chemistry in the development of α-emitting radiopharmaceuticals will continue to grow. However, the click chemistry-based pre-targeting approach for TAT remains underdeveloped despite its many advantages. Fortunately, the promising aspects are driving ongoing research focused on optimizing component stability within the complex tumor microenvironment, ensuring efficient clearance of pre-targeting agents, and achieving rapid in vivo reaction kinetics. These efforts aim to minimize immune responses and reduce radiation exposure to healthy organs. An accompanying advantage is the possibility of separately optimizing the tumor uptake of the pre-targeting vector, including the development of mechanisms for the removal of its unbound fraction from the blood circulation. At the same time, the need to synthesize two products (a targeting molecule and a radiocomplex) as well as optimize dosing protocols for both components may appear less attractive for clinical implementation, which can explain the rather slow progress of pre-targeted α-radiotherapy. Clearly, the path to widespread clinical application of click chemistry for α-radiotherapy requires addressing both technical and translational challenges. This may be achieved through continued acquisition of preclinical data demonstrating the value of this modality for cancer treatment.

## Data Availability

No new data were created or analyzed in this study. Data sharing is not applicable to this article.

## References

[1] Artigas C., Mileva M., Flamen P., Karfis I. (2021). Targeted radionuclide therapy: An emerging field in solid tumours. Curr. Opin. Oncol..

[2] Liepe K., Runge R., Kotzerke J. (2005). Systemic radionuclide therapy in pain palliation. Am. J. Hosp. Palliat. Med..

[3] Helal M., Dadachova E. (2018). Radioimmunotherapy as a Novel Approach in HIV, Bacterial, and Fungal Infectious Diseases. Cancer Biother. Radiopharm..

[4] Kassis A.I. (2008). Therapeutic radionuclides: Biophysical and radiobiologic principles. Semin. Nucl. Med..

[5] Dekempeneer Y., Keyaerts M., Krasniqi A., Puttemans J., Muyldermans S., Lahoutte T., D’huyvetter M., Devoogdt N. (2016). Targeted alpha therapy using short-lived alpha-particles and the promise of nanobodies as targeting vehicle. Expert. Opin. Biol. Ther..

[6] Nelson B.J.B., Andersson J.D., Wuest F. (2021). Targeted Alpha Therapy: Progress in Radionuclide Production, Radiochemistry, and Applications. Pharmaceutics.

[7] Ferrier M.G., Radchenko V., Wilbur D.S. (2019). Radiochemical aspects of alpha emitting radionuclides for medical application. Radiochim. Acta.

[8] Poty S., Francesconi L.C., McDevitt M.R., Morris M.J., Lewis J.S. (2018). alpha-Emitters for Radiotherapy: From Basic Radiochemistry to Clinical Studies-Part 1. J. Nucl. Med..

[9] Ostuni E., Taylor M.R.G. (2022). Commercial and business aspects of alpha radioligand therapeutics. Front. Med..

[10] Stokke C., Kvassheim M., Blakkisrud J. (2022). Radionuclides for Targeted Therapy: Physical Properties. Molecules.

[11] Pallares R.M., Abergel R.J. (2022). Development of radiopharmaceuticals for targeted alpha therapy: Where do we stand?. Front. Med..

[12] De Kruijff R.M., Wolterbeek H.T., Denkova A.G. (2015). A Critical Review of Alpha Radionuclide Therapy—How to Deal with Recoiling Daughters?. Pharmaceuticals.

[13] Fedorchenko D., Alani S. (2023). Simulation of particle release for Diffusing Alpha-Emitters Radiation Therapy. Appl. Radiat. Isot..

[14] Rodriguez C., Delaney S., Sarrett S.M., Keinänen O.M., Zeglis B.M. (2022). Antibody Engineering for Nuclear Imaging and Radioimmunotherapy. J. Nucl. Med..

[15] Verhoeven M., Seimbille Y., Dalm S.U. (2019). Therapeutic Applications of Pretargeting. Pharmaceutics.

[16] Boerman O.C., van Schaijk F.G., Oyen W.J.G., Corstens F.H.M. (2003). Pretargeted Radioimmunotherapy of Cancer: Progress Step by Step. J. Nucl. Med..

[17] Beishenaliev A., Loke Y.L., Goh S.J., Geo H.N., Mugila M., Misran M., Chung L.Y., Kiew L.V., Roffler S., Teo Y.Y. (2023). Bispecific antibodies for targeted delivery of anti-cancer therapeutic agents: A review. J. Control. Release.

[18] Edelmann M.R., Sladojevich F., Husbands S.M., Otteneder M.B., Blagbrough I.S. (2024). A Brief Review of Radiolabelling Nucleic Acid-Based Molecules for Tracking and Monitoring. J. Label. Compd. Radiopharm..

[19] Mitry M.M.A., Greco F., Osborn H.M.I. (2023). In Vivo Applications of Bioorthogonal Reactions: Chemistry and Targeting Mechanisms. Chem. Eur. J..

[20] Live Chart of Nuclides. https://www-nds.iaea.org/relnsd/vcharthtml/VChartHTML.html.

[21] Van Laere C., Koole M., Deroose C.M., de Voorde M.V., Baete K., Cocolios T.E., Duchemin C., Ooms M., Cleeren F. (2024). Terbium radionuclides for theranostic applications in nuclear medicine: From atom to bedside. Theranostics.

[22] Munekane M., Fuchigami T., Ogawa K. (2024). Recent advances in the development of ^225^Ac- and ^211^At-labeled radioligands for radiotheranostics. Anal. Sci..

[23] Mourtada F., Tomiyoshi K., Sims-Mourtada J., Mukai-Sasaki Y., Yagihashi T., Namiki Y., Murai T., Yang D.J., Inoue T. (2023). Actinium-225 Targeted Agents: Where Are We Now?. Brachytherapy.

[24] Dhiman D., Vatsa R., Sood A. (2022). Challenges and opportunities in developing Actinium-225 radiopharmaceuticals. Nuclear Med. Commun..

[25] Ahenkorah S., Cassells I., Deroose C.M., Cardinaels T., Burgoyne A.R., Bormans G., Ooms M., Cleeren F. (2021). Bismuth-213 for Targeted Radionuclide Therapy: From Atom to Bedside. Pharmaceutics.

[26] Juzeniene A., Stenberg V.Y., Bruland Ø.S., Revheim M.-E., Larsen R.H. (2023). Dual targeting with ^224^Ra/^212^Pb-conjugates for targeted alpha therapy of disseminated cancers: A conceptual approach. Front. Med..

[27] Trencsényi G., Csikos C., Képes Z. (2024). Targeted Radium Alpha Therapy in the Era of Nanomedicine: In Vivo Results. Int. J. Mol. Sci..

[28] Wick R.R., Gössner W. (1993). History and current uses of ^224^Ra in ankylosing spondylitis and other diseases. Environ. Int..

[29] Gupta N., Devgan A., Bansal I., Olsavsky T.D., Li S., Abdelbaki A., Kumar Y. (2017). Usefulness of radium-223 in patients with bone metastases. Bayl. Univ. Med. Cent. Proc..

[30] Sathekge M., Bruchertseifer F., Vorster M., Lawal I.O., Knoesen O., Mahapane J., Davis C., Mdlophane A., Maes A., Mokoala K. (2022). 3mCRPC Patients Receiving ^225^Ac-PSMA-617 Therapy in the Post-Androgen Deprivation Therapy Setting: Response to Treatment and Survival Analysis. J. Nucl. Med..

[31] Sathekge M., Bruchertseifer F., Knoesen O., Reyneke F., Lawal I., Lengana T., Davis C., Mahapane J., Corbett C., Vorster M. (2019). ^225^Ac-PSMA-617 in chemotherapy-naive patients with advanced prostate cancer: A pilot study. Eur. J. Nucl. Med. Mol. Imaging.

[32] Kratochwil C., Bruchertseifer F., Giesel F.L., Weis M., Verburg F.A., Mottaghy F., Kopka K., Apostolidis C., Haberkorn U., Morgenstern A. (2016). ^225^Ac-PSMA-617 for PSMA-Targeted alpha-Radiation Therapy of Metastatic Castration-Resistant Prostate Cancer. J. Nucl. Med..

[33] Ballal S., Yadav M.P., Bal C., Sahoo R.K., Tripathi M. (2020). Broadening horizons with ^225^Ac-DOTATATE targeted alpha therapy for gastroenteropancreatic neuroendocrine tumour patients stable or refractory to ^177^Lu-DOTATATE PRRT: First clinical experience on the efficacy and safety. Eur. J. Nucl. Med. Mol. Imaging.

[34] Królicki L., Kunikowska J., Bruchertseifer F., Koziara H., Królicki B., Jakuciński M., Pawlak D., Rola R., Morgenstern A., Rosiak E. (2020). ^225^Ac- and ^213^Bi-Substance P Analogues for Glioma Therapy. Semin. Nucl. Med..

[35] Cordier D., Krolicki L., Morgenstern A., Merlo A. (2016). Targeted Radiolabeled Compounds in Glioma Therapy. Semin. Nucl. Med..

[36] Kratochwil C., Giesel F.L., Bruchertseifer F., Mier W., Apostolidis C., Boll R., Murphy K., Haberkorn U., Morgenstern A. (2014). ^213^Bi-DOTATOC receptor-targeted alpha-radionuclide therapy induces remission in neuroendocrine tumours refractory to beta radiation: A first-in-human experience. Eur. J. Nucl. Med. Mol. Imaging.

[37] Kratochwil C., Flechsig P., Lindner T., Abderrahim L., Altmann A., Mier W., Adeberg S., Rathke H., Röhrich M., Winter H. (2019). ^68^Ga-FAPI PET/CT: Tracer Uptake in 28 Different Kinds of Cancer. J. Nucl. Med..

[38] Lindner T., Loktev A., Altmann A., Giesel F., Kratochwil C., Debus J., Jäger D., Mier W., Haberkorn U. (2018). Development of Quinoline-Based Theranostic Ligands for the Targeting of Fibroblast Activation Protein. J. Nucl. Med..

[39] Watabe T., Liu Y., Kaneda-Nakashima K., Shirakami Y., Lindner T., Ooe K., Toyoshima A., Nagata K., Shimosegawa E., Haberkorn U. (2020). Theranostics Targeting Fibroblast Activation Protein in the Tumor Stroma: ^64^Cu- and ^225^Ac-Labeled FAPI-04 in Pancreatic Cancer Xenograft Mouse Models. J. Nucl. Med..

[40] Assadi M., Rekabpour S.J., Jafari E., Divband G., Nikkholgh B., Amini H., Kamali H., Ebrahimi S., Shakibazad N., Jokar N. (2021). Feasibility and Therapeutic Potential of ^177^Lu–Fibroblast Activation Protein Inhibitor–46 for Patients With Relapsed or Refractory Cancers: A Preliminary Study. Clin. Nucl. Med..

[41] Liu Y., Watabe T., Kaneda-Nakashima K., Shirakami Y., Naka S., Ooe K., Toyoshima A., Nagata K., Haberkorn U., Kratochwil C. (2022). Fibroblast activation protein targeted therapy using [^177^Lu]FAPI-46 compared with [^225^Ac]FAPI-46 in a pancreatic cancer model. Eur. J. Nucl. Med. Mol. Imaging.

[42] Lepareur N., Ramée B., Mougin-Degraef M., Bourgeois M. (2023). Clinical Advances and Perspectives in Targeted Radionuclide Therapy. Pharmaceutics.

[43] Mdanda S., Mdlophane A., Ndlovu H., Ramonaheng K., Qebetu M., Mahapane J., Kgatle M., Mzizi Y., Sebatana R., Cele Z.E.D. (2023). Targeted Alpha Therapy in Cancer Management: Therapeutic Prospects of Nuclear Medicine in Oncology. Interdisciplinary Cancer Research.

[44] Miederer M., Benešová-Schäfer M., Mamat C., Kästner D., Pretze M., Michler E., Brogsitter C., Kotzerke J., Kopka K., Scheinberg D.A. (2024). Alpha-Emitting Radionuclides: Current Status and Future Perspectives. Pharmaceuticals.

[45] Albertsson P., Bäck T., Bergmark K., Hallqvist A., Johansson M., Aneheim E., Lindegren S., Timperanza C., Smerud K., Palm S. (2023). Astatine-211 based radionuclide therapy: Current clinical trial landscape. Front. Med..

[46] Jurcic J.G. (2020). Targeted Alpha-Particle Therapy for Hematologic Malignancies. Semin. Nucl. Med..

[47] Price E.W., Orvig C. (2014). Matching chelators to radiometals for radiopharmaceuticals. Chem. Soc. Rev..

[48] Baranyai Z., Tircsó G., Rösch F. (2020). The Use of the Macrocyclic Chelator DOTA in Radiochemical Separations. Eur. J. Inorg. Chem..

[49] Peters J.A., Djanashvili K., Geraldes C.F.G.C., Platas-Iglesias C. (2020). The chemical consequences of the gradual decrease of the ionic radius along the Ln-series. Coord. Chem. Rev..

[50] Thiele N.A., Wilson J.J. (2018). Actinium-225 for Targeted α Therapy: Coordination Chemistry and Current Chelation Approaches. Cancer Biother. Radiopharm..

[51] Deal K.A., Davis I.A., Mirzadeh S., Kennel S.J., Brechbiel M.W. (1999). Improved in Vivo Stability of Actinium-225 Macrocyclic Complexes. J. Med. Chem..

[52] Ivanov A.S., Simms M.E., Bryantsev V.S., Benny P.D., Griswold J.R., Delmau L.H., Thiele N.A. (2022). Elucidating the coordination chemistry of the radium ion for targeted alpha therapy. Chem. Commun..

[53] Matazova E.V., Egorova B.V., Zubenko A.D., Pashanova A.V., Mitrofanov A.A., Fedorova O.A., Ermolaev S.V., Vasiliev A.N., Kalmykov S.N. (2023). Insights into Actinium Complexes with Tetraacetates—AcBATA versus AcDOTA: Thermodynamic, Structural, and Labeling Properties. Inorg. Chem..

[54] Deblonde G.J.P., Lohrey T.D., Booth C.H., Carter K.P., Parker B.F., Larsen Å., Smeets R., Ryan O.B., Cuthbertson A.S., Abergel R.J. (2018). Solution Thermodynamics and Kinetics of Metal Complexation with a Hydroxypyridinone Chelator Designed for Thorium-227 Targeted Alpha Therapy. Inorg. Chem..

[55] Pham T.A., Xu J., Raymond K.N. (2014). A Macrocyclic Chelator with Unprecedented Th^4+^ Affinity. J. Am. Chem. Soc..

[56] Woods J.J., Cosby A.G., Wacker J.N., Aguirre Quintana L.M., Peterson A., Minasian S.G., Abergel R.J. (2023). Macrocyclic 1,2-Hydroxypyridinone-Based Chelators as Potential Ligands for Thorium-227 and Zirconium-89 Radiopharmaceuticals. Inorg. Chem..

[57] Szucs Z., van Rooyen J., Zeevaart J.R. (2009). Recoil effect on beta-decaying in vivo generators, interpreted for ^103^Pd/^103m^Rh. Appl. Radiat. Isot..

[58] Hu A., Wilson J.J. (2022). Advancing Chelation Strategies for Large Metal Ions for Nuclear Medicine Applications. Acc. Chem. Res..

[59] Spicer C.D., Pashuck E.T., Stevens M.M. (2018). Achieving Controlled Biomolecule–Biomaterial Conjugation. Chem. Rev..

[60] McDevitt M.R., Ma D., Simon J., Frank R.K., Scheinberg D.A. (2002). Design and synthesis of ^225^Ac radioimmunopharmaceuticals. Appl. Radiat. Isot..

[61] Borchardt P.E., Yuan R.R., Miederer M., McDevitt M.R., Scheinberg D.A. (2003). Targeted actinium-225 in vivo generators for therapy of ovarian cancer. Cancer Res..

[62] Maguire W.F., McDevitt M.R., Smith-Jones P.M., Scheinberg D.A. (2014). Efficient 1-step radiolabeling of monoclonal antibodies to high specific activity with ^225^Ac for α-particle radioimmunotherapy of cancer. J. Nucl. Med..

[63] Poty S., Membreno R., Glaser J.M., Ragupathi A., Scholz W.W., Zeglis B.M., Lewis J.S. (2018). The inverse electron-demand Diels–Alder reaction as a new methodology for the synthesis of ^225^Ac-labelled radioimmunoconjugates. Chem. Commun..

[64] Denk C., Wilkovitsch M., Aneheim E., Herth M.M., Jensen H., Lindegren S., Mikula H. (2019). Multifunctional Clickable Reagents for Rapid Bioorthogonal Astatination and Radio-Crosslinking. ChemPlusChem.

[65] Reissig F., Bauer D., Zarschler K., Novy Z., Bendova K., Ludik M.-C., Kopka K., Pietzsch H.-J., Petrik M., Mamat C. (2021). Towards Targeted Alpha Therapy with Actinium-225: Chelators for Mild Condition Radiolabeling and Targeting PSMA—A Proof of Concept Study. Cancers.

[66] Bauer D., Cornejo M.A., Hoang T.T., Lewis J.S., Zeglis B.M. (2023). Click Chemistry and Radiochemistry: An Update. Bioconj. Chem..

[67] Peppicelli S., Calorini L., Bianchini F., Papucci L., Magnelli L., Andreucci E. (2025). Acidity and hypoxia of tumor microenvironment, a positive interplay in extracellular vesicle release by tumor cells. Cell. Oncol..

[68] Lammers T. (2024). Nanomedicine Tumor Targeting. Adv. Mater..

[69] Reardan D.T., Meares C.F., Goodwin D.A., McTigue M., David G.S., Stone M.R., Leung J.P., Bartholomew R.M., Frincke J.M. (1985). Antibodies against metal chelates. Nature.

[70] Berton C., Klingler S., Prytuliak S., Holland J.P. (2024). New tactics in the design of theranostic radiotracers. npj Imaging.

[71] Huang Z., Hu Y., Yang Y., Huang W., Wang Y., Ye D. (2022). Recent Advances in Pretargeted Imaging of Tumors in Vivo. Anal. Sens..

[72] Staudt M., Herth M.M. (2023). Clearing and Masking Agents in Pretargeting Strategies. Pharmaceuticals.

[73] Goodwin D.A., Mears C.F., McTigue M., David G.S. (1986). Monoclonal antibody hapten radiopharmaceutical delivery. Nuclear Med. Commun..

[74] Cheal S.M., Chung S.K., Vaughn B.A., Cheung N.-K.V., Larson S.M. (2022). Pretargeting: A Path Forward for Radioimmunotherapy. J. Nucl. Med..

[75] Poty S., Carter L.M., Mandleywala K., Membreno R., Abdel-Atti D., Ragupathi A., Scholz W.W., Zeglis B.M., Lewis J.S. (2019). Leveraging Bioorthogonal Click Chemistry to Improve ^225^Ac-Radioimmunotherapy of Pancreatic Ductal Adenocarcinoma. Clin. Cancer Res..

[76] Rossin R., Renart Verkerk P., van den Bosch S.M., Vulders R.C.M., Verel I., Lub J., Robillard M.S. (2010). In Vivo Chemistry for Pretargeted Tumor Imaging in Live Mice. Angew. Chem. Int. Ed..

[77] Stéen E.J.L., Jørgensen J.T., Denk C., Battisti U.M., Nørregaard K., Edem P.E., Bratteby K., Shalgunov V., Wilkovitsch M., Svatunek D. (2021). Lipophilicity and Click Reactivity Determine the Performance of Bioorthogonal Tetrazine Tools in Pretargeted In Vivo Chemistry. ACS Pharmacol. Transl. Sci..

[78] Timperanza C., Jensen H., Bäck T., Lindegren S., Aneheim E. (2023). Pretargeted Alpha Therapy of Disseminated Cancer Combining Click Chemistry and Astatine-211. Pharmaceuticals.

